# Spatial disorientation and countermeasure training for next-generation aircraft: a review and recommendations

**DOI:** 10.3389/fphys.2026.1830128

**Published:** 2026-08-10

**Authors:** John Jong-Jin Kim, Ramy Kirollos

**Affiliations:** Defense Research and Development Canada, Toronto Research Center, Toronto, ON, Canada

**Keywords:** military aviation, spatial disorientation, training, vestibular illusions, visual-vestibular sensory integration

## Abstract

Spatial disorientation (SD) is the most common cause of human error in military aircraft incident reports over the last few decades. Vestibular SD typically occurs when the vestibular (balance) system cannot distinguish between acceleration from gravity and from the aircraft. Current and next-generation fighter aircraft (e.g., F-35) expose pilots to unprecedented acceleration forces, possibly amplifying SD frequency and severity. Therefore, research investigating SD and countermeasures in modern fighter aircraft is becoming increasingly important. The main objective of this review paper was to assess modern technologies that can be used in counter-SD training and provide directions for future research on SD training. To accomplish this objective, we reviewed a total of 118 books, technical reports, and scientific papers to examine the current state of knowledge in SD and countermeasures used in military aviation. Past literature mostly focused on the subjective pilot experience without considering the human sensory system as the root cause. In addition, studies investigating modern technologies (e.g., virtual reality head-mounted display) in SD countermeasure training were limited. In this review, we define and evaluate the common SD illusions and their causes based on the available scientific literature. Then, we examine successful contemporary SD countermeasure training methods and tools currently used. Finally, we provide recommendations for future SD research focusing on vestibular SD countermeasure training given recent technological advancements covered. Our recommendations include the exploration of various combinations of artificial vestibular stimulation, virtual reality, and centrifuge as viable SD training methods. We also provide potential solutions to evaluate SD countermeasures effectively and objectively.

## 1 Introduction

A human’s perception of their orientation relative to the world involves integrating multi-sensory signals from various sensory organs (e.g., visual, auditory, somatosensory, and vestibular senses; Bos et al., 2003). This is important to effectively and safely navigate the world. The Earth’s gravitational force is an important acceleration cue for orientation reference estimates acting on the vestibular organs in the inner ear (Gentaz and Hatwell, 1996). But the accelerations due to gravity and those due to motion are equivalent per their physical nature (Einstein’s equivalence principle) and are therefore indistinguishable to the vestibular apparatus. Additionally, travelling at constant velocity and being stationary are also indistinguishable to the vestibular apparatus due to its inertial properties (Lishman and Lee, 1973). Because vestibular signals are ambiguous, a person can lose their sense of position, orientation, and motion in reference to the gravitational upright (or vertical) when visual and proprioceptive cues are insufficient or absent. This phenomenon is called spatial disorientation (SD; Davis et al., 2008; Gradwell and Rainford, 2016; Riski and Wibawanti, 2024; Sánchez-Tena et al., 2018).

SD while walking can increase the risk of falling, which can have serious life-altering consequences for the elderly (Alamgir et al., 2012; Spaniolas et al., 2010). But it does not pose a significant risk among healthy young individuals. In contrast, SD in pilots operating aircraft can be fatal as it imposes impairments in sensory perception during high-level cognitive performance (Fudali-Czyż et al., 2024; Gresty and Golding, 2009; Gresty et al., 2008; Stróżak et al., 2018; Webb et al., 2012), leading to erroneous perception of the aircraft’s disposition and attitude. This is of critical concern as many past reports named SD as the most common cause of human error-related aircraft accidents over the last few decades (Bles, 2008; Cheung, 2013; Heinle and Ercoline, 2002; Holmes et al., 2003; Lawson et al., 2017). For example, Gaydos et al. (2012) reported that SD contributed to 100 Class A-C rotary-wing (RW) flight mishaps in the U.S. between 2002-2011, with 22% resulting in fatalities. **Table 1** lists the mishap classifications criteria defined by U.S. Army Combat Readiness Center (USACRC) and their outcome (Curry and Lee, 2024). Heinle and Ercoline (2002) also reported the economic consequences of SD incidents which cost the United States Air Force (USAF) approximately $557M USD due to the loss of aircraft between 1990–1999.

**Table 1:** USACRC mishap classification definitions adapted from Curry and Lee (2024).

Class	Definition
A	An Army manned aircraft is destroyed, missing, or abandoned; or an injury and/or occupational illness results in a fatality or permanent total disability.
B	An injury and/or occupational illness results in permanent partial disability, or when 3 or more personnel are hospitalized as inpatients as the result of a single occurrence.
C	A nonfatal injury and/or occupational illness that causes 1 or more days away from work or training beyond the day or shift on which it occurred or disability at any time (that does not meet the definition of Class A or B and is a lost time case).
D	A nonfatal injury or illness resulting in restricted work, transfer to another job, medical treatment greater than first aid.
E	No injury or illness.

Various SD mitigation strategies have been implemented over the decades. For example, SD training and education programs around the world commonly expose student pilots to vestibular illusions (e.g., *Coriolis*) using a rotating chair (e.g., the Bárány chair) for ground-based SD demonstrations. Yet the overall SD rates do not seem to have decreased over time based on reports by Heinle and Ercoline (2002) and Bles (2008). Another report, covering a more recent time period from Gaydos et al. (2012) suggested that the SD-related mishaps decreased in the military RW community between 2003-2011. However, in general aviation (i.e., civil aviation that excludes commercial airliners and military flights), the reported SD mishaps increased between 1981-2016 (Newman and Rupert, 2020). Overall, these reports show that SD remains a significant risk to aircrews. Hence, more rigorous research needs to be conducted to counter SD (Cheung, 2013; Gaydos et al., 2012; Gibb et al., 2011).

Current and next-generation fighter aircraft like the F-35 and Gripen can expose pilots to intense and unprecedented physical stress. These aircraft are capable of operating at up to 50,000 ft (~ 15.2 km), fly at supersonic speed (e.g., Mach 1.6 for F-35) and accelerate to 9 G (generally measured relative to Earth’s gravity in g-force, i.e., G; Försvarets materielverk, 2022; Lockheed Martin Corporation, 2023). The human body is not accustomed to the stress involved with flying these aircraft. Moreover, health and cognitive outcomes associated with flying these aircraft are still largely unknown. SD generally arises from the discrepancies between the human sensory systems as well as cognitive function involved in executing a task (Heinle and Ercoline, 2002). The extremely high speeds and g-forces capable of these fighter aircraft, and the psychophysical stress they can impose on pilots may increase SD occurrence and intensity.

### 1.1 The vestibular system’s contribution to SD

To understand the possible contribution of new generation fighter aircraft on aircrew SD, we must first describe how people perceive their position, orientation and motion of their body in the world. Visual cues like optic flow and the horizon are generally reliable for judging our body’s position relative to Earth. Other physical cues interpreted by interoceptive signals (i.e., sensations from within the body) to self-motion can be ambiguous. This is because they are mostly suited to detect acceleration from inertia of the fluid and organs in the body (Teaford et al., 2022), like the vestibular system’s endolymph fluid (Day and Fitzpatrick, 2005; Warren and Wertheim, 1990).

**Figure 1:**
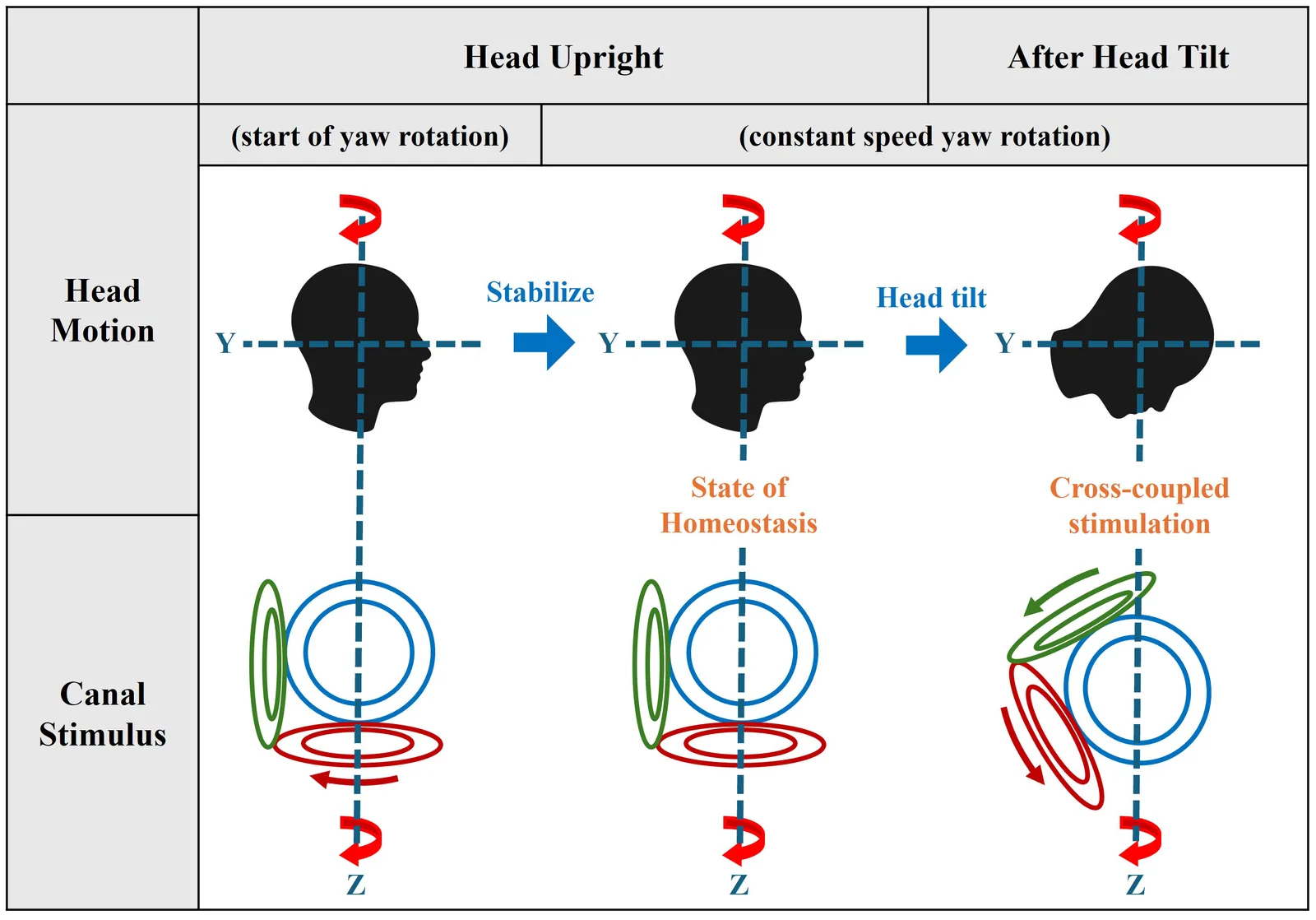
An example of *Coriolis* effect. During a constant yaw rotation (along the z-axis), SCCs reach the state of homeostasis (i.e., equilibrium). After a sudden forward head tilt, the change in the canal orientations (relative to the y- and z-axes) causes cross-coupled stimulation (on the PC and LC). This *Coriolis* effect leads to a false sensation of roll rotation. The SCCs are simplified here and are represented with blue (AC) green (PC) and red (LC) rings.

The vestibular system comprises two main organs in each ear: the semi-circular canals (SCCs) fordetecting rotational acceleration (pitch, roll and yaw) and the otolith organs for detecting translational acceleration (forward/backward, up/down and left/right). These organs rely on inertial properties of the endolymph fluid acting on the cupular membranes in the SCCs and on the otolithic membranes in the otolith organs’ hair cells to trigger neural firing in the brain for perceiving direction and magnitude of acceleration of the head (see Herdman and Clendaniel, 2014 for details). The SCCs are comprised of three canals: the anterior (superior) canal (AC), posterior canal (PC), and lateral (horizontal) canals (LC). The SCCs respond to the rotational acceleration of the head (Bos et al., 2003; Day and Fitzpatrick, 2005; Warren and Wertheim, 1990) in the corresponding plane of each canal (Baloh, 2003; Rabbitt, 2019). The LC is located at a 30° angle below Earth’s horizontal when the head is upright (Khan and Chang, 2013). The SC and PC are both positioned vertically at 90° from each other and at approximately 45° relative to one’s frontal plane. The SCCs in the left and right ear are orientated in a mirror-image arrangement, resulting in the coplanar pairing of the canals between ears (refer to Figures 1–4C in Baloh, 2003). Therefore, the perception of pitch, roll, and yaw of the head results from the integration of signals across the six canals in both ears. This orthogonal configuration of the SCCs allows for precise detection of angular acceleration of the head.

The otolith organs are comprised of the utricle and the saccule. They detect linear acceleration of the head. The utricle is aligned on roughly the same plane as the horizontal canal at a 30° angle from horizontal, and the saccule is positioned vertically when the head is upright. This configuration in part allows otolith organs to detect linear acceleration in the horizontal (utricle) and vertical (saccule) planes. The otoliths cannot distinguish linear acceleration caused by head translation from the acceleration caused by gravity (Angelaki and Cullen, 2008). The vestibular signals from both the otolith organs and SCCs have generally been found to be integrated together (Merfeld et al., 2005) and with visual and proprioceptive cues in the cortex (Harris, 2022; Hlavacka et al., 1996). The integrations are performed in a statistically optimal fashion, reducing perceptual ambiguities (some papers demonstrating this include; Campos et al., 2014; Kirollos and Herdman, 2023a, 2023b; ter Horst et al., 2015). Though there has been accounts of visual (Lishman and Lee, 1973; McManus and Harris, 2023) and vestibular (Butler et al., 2010; Harris et al., 2000) cue dominance.

Visual-vestibular cue conflict is one of the main causes of SD. It is defined as an incongruence between visual and vestibular percepts. Visual-vestibular conflict can arise in degraded visual environments (DVE). An example of a DVE causing SD is when dust or snow is blown by heavy wind from a RW aircraft (i.e., brown- or white-outs), causing the pilot to experience visually induced-illusory self-motion known as ‘*vection*’ (Howard and Howard, 1994). This illusion, caused by the global visual motion of the dust or snow relative to the pilot, can make them feel like they are spiraling despite being stationary (i.e., the aircraft hovering). Night flying is another prominent example of a DVE leading to increased risk of SD (Lyons et al., 2006). Pilots have reported that SD incidents at night are more severe than during the day (Takada et al., 2009).

### 1.2 Paper contributions and objectives

Most studies on SD in aviation report the illusions by describing the pilots' experience and their frequencies (e.g., Bles, 2008; Gibb et al., 2011; Lewkowicz and Biernacki, 2020). Very few studies we found considered the root causes of the SD illusions from the human sensory systems. The SD illusions in the literature are typically categorized as visual or vestibular illusions. But to our knowledge, no past reviews on SD focused on the vestibular system. In addition, the literature on SD in aviation is generally more than a decade old. Hence, there are few empirical studies on SD countermeasures that implement new modern technologies (e.g., artificial vestibular stimulation and virtual reality).

In this review paper, we provide a contemporary account of the state of SD research in the healthy vestibular system in the aviation context. We start by listing the names and the prevalence of common SD illusions. We define and evaluate their causes with a main focus on vestibular illusions. Next, we examine success rates of established SD countermeasures found throughout the available literature. Then we survey new technologies that may be promising for improving and modernizing SD countermeasure training and recommend new possible advancements to current SD training programs. Finally, we provide directions for future research.

## 2 Background

For this narrative review, the literature search was conducted by the first author using the ‘snowball’ search method. Relevant peer-reviewed journal and conference papers, military reports and textbooks on the topic of SD were searched on Google Scholar, PubMed, Aerospace Medical Association, Canadian Defence Information Database and Defense Technical Information Center between October 8^th^, 2025 and February 5^th^, 2026. To broaden our search further, we also searched on Scopus and Web of Science between April 14^th^, 2026 and April 20^th^, 2026. Searches were performed with these specific keywords: “spatial disorientation” in combination with either “aviation”, “cause”, “countermeasure”, “training”, “vestibular stimulation”, or “centrifuge”. The publications returned by keyword searches were evaluated for their relevance to SD in military aviation and countermeasure training. The exclusion criteria applied to the returned literature was as follows: 1) research not explicitly related to SD in aviation (e.g., SD occurring on the ground and in space, SD caused by medical conditions, or research that solely measured general human spatial perception), 2) research not involving human factors (e.g., countermeasures using technology, algorithms, or artificial intelligence without human pilot involvement) and 3) animal studies (i.e., with non-human subjects). After reviewing the literature based on keyword searches and exclusionary criteria, other relevant works were identified from backward and forward reference searches for relevant publications.

As a result, a total of 118 published scholarly sources in English and Korean were reviewed for this paper: 4 books, 24 technical reports (for the organizations including the Departments of Transportation, Militaries, and the North Atlantic Treaty Organization Science and Technology Organization; NATO STO), 15 review papers, 70 original research papers (survey, analytical, experimental and modeling) and 5 other types of articles (commentaries, opinions and letters).

### 2.1 SD and its prevalence in aviation

In a NATO Research and Technology Organization (RTO) report, Bles (2008) reported SD-related mishaps in NATO partner armed forces, available SD training solutions and recommendations. The SD-related mishaps between the 1960s to early 2000s from the report are summarized in **Table 2**. A recent example of an SD-related accident was from the Royal Canadian Air Force (RCAF). On June 19^th^ 2023, during an Advanced Night Test of the Tactical First Officer Course, aircraft CH147310 crashed into the Ottawa River (Canadian Armed Forces, 2024). Investigators concluded that SD due to unperceived aircraft acceleration and the environmental conditions caused the accident, resulting in the loss of two pilots and the destruction of the helicopter. Two Flight Engineers survived with minor injuries.

**Table 2:** SD-related accidents summarized from the literature (Bles, 2008; Braithwaite et al., 1998a; Bushby et al., 2004; Cheung et al., 1995; Curry and McGhee, 2007; Hartzell, 1979; Lyons et al., 2006).

Military branch	Time period	Reported SD-related mishaps
North America		
US Air Force (USAF)	1993 – 2002	13.2% of all Class A accidents; 100 accidents, loss of 19 lives and 25 aircraft
	2000 – 2004	11% of all crashes with fatality rate of 69%
US Army	1987 – 1995	30% of all Class A – C
	2000 – 2005	37% of all Class A – C
Royal Canadian Air Force (RCAF)	1968 – 1978	12 accidents, loss of 8 lives and 10 aircraft
	1982 – 1992	23% of all Class A accidents
Europe		
Czech Air Force	1991 – 2005	23% of all Class A accidents; accounted for 50% of all fatalities
Italian Air Force (ITAF)	1993 – 2004	19% of all Class A accidents; accounted for 33% of all fatalities
Royal Netherlands Air Force (RNLAF)	1980 – 2005	16.7% of all Class A accidents
Swedish Air Force	1986 – 2005	16% (11/42) of all Class A accidents
French Air Force	2000 – 2004	4% of all Class A accidents
Bundeswehr (the armed forces of the Federal Republic of Germany)	1995 – 2006	5% of all Class A accidents
Helenic Air Force (Greece)	1990 – 2005	56.5% (35/62) of major accidents (i.e., loss of life and/or aircraft)
United Kingdom Royal Air Force (RAF)	1983 – 1992	24.7% of all aircraft accidents
	1993 – 2002	33.0% of all aircraft accidents

SD during flight poses a threat in civil aviation, too. For example, accident summaries reported in the air transportation occurrence data (Transportation Safety Board of Canada, 1995) mentioned disorientation in 24 of the Class A-C accidents between 1976-1995. Newman and Rupert (2020) reviewed various transportation safety databases and identified 94 SD mishaps between 1981–2016 in general aviation. In the National Transportation Safety Board aviation accident database, SD accidents accounted for 9.9% of the fatal accidents between 1983-1991 (Mortimer, 1995) and 7.4% between 2003-2021 (Baumgartner et al., 2025). The Flight Safety Foundation estimates 10% of all commercial aviation accidents can be attributed to SD (Kowalski, 2024).

Gibb et al. (2011) argued that SD incidents are likely under-reported. They estimate that 25-33% of all aircraft incidents are in part caused by SD. To determine whether a given incident was caused by SD, the investigation needs to take the pilot’s account of events. However, the high fatality rate in the SD accidents leaves many questions unanswered (Gibb et al., 2011; Veronneau and Evans, 2004). Another reason for under-reporting SD is that there can sometimes be resistance among the investigators to include human factors such as SD as the cause of an incident, partially due to the difficulty quantifying SD and to also avoid possibly ‘blaming’ aircrew.

### 2.2 Types of illusions causing SD

Past reports have categorized SD incidents into four categories: visual illusion – SD due to misinterpreted visual cues, body sense illusion – SD due to misinterpreted non-visual cues (in most cases, vestibular cues), displays – SD related to using displays (e.g., flight instruments), and ‘other’. Lewkowicz and Biernacki (2020) listed the pilot SD experiences and causes reported in Polish Armed Forces (PAF), comparing them to the survey results in the United Kingdom’s Royal Air Force (RAF; Holmes et al., 2003), the United States Air Force (USAF; Matthews et al., 2002) and the Royal Netherlands Air Force (RNLAF; Pennings et al., 2020). **Table 3** lists the SD illusions reported and their prevalence.

**Table 3:** Examples of SD illusions and frequencies from surveys conducted between 1970–2017 with pilots and aircrews in various militaries.

Type	Illusion	Respondents [%]			
		PAF *(N = 176)*	RAF *(N = 78)*	USAF *(N = 2,582)*	RNLAF *(N = 368)*
Visual Illusions	Loss of horizon (atmospheric conditions)	81	82	69	70
	Sloping horizon (or false horizon)	55	75	66	76
	Night approach (or Black hole illusion)	36	60	58	58
	Misleading altitude cues from ground texture (e.g., over flat ward, small trees)	50	79	50	66
	Misjudgment of position in night formation	29	37	38	38
	Autokinesis	43	43	37	45
	Loss of horizon (brown-out/white-out/spray out)	45	56	33	45
	Vection (or False sense of yaw)	36	20	31	36
Body Sense Illusions	Leans	41	92	76	67
	Tumbling sensation (Coriolis)	41	66	61	57
	False sense of pitching up	14	34	44	30
	Elevator illusion	34	35	37	39
	Graveyard spiral	43	43	32	48
	G-excess	15	33	36	37
	False sense of pitching down	9	28	36	29
	False sense of inversion	34	18	23	27
	Undetected drift	18	55	6	38
	Graveyard spin	36	7	9	8
Displays	Roll-reversal error	37	31	23	24
	Caused by Head-Up Display (HUD) use	9	13	10	15
	Caused by Forward Looking Infra-Red (FLIR) use	4	11	9	27
	Caused by Helmet-Mounted Displays (HMD) use	3	2	2	28
Other	Poor crew co-ordination	29	50	40	21
	Giant hand	55	31	38	17
	Feeling of detachment (high altitude)	37	17	11	12

The frequencies of each illusion, measured based on the number of respondents that reported experiencing the illusion (%), are adapted from Lewkowicz and Biernacki (2020).

These SD illusions have various symptoms and causes, many of which cannot be interpreted from their names alone. The following sections provide details and explanations for some of the common SD illusions listed in **Table 3**.

#### 2.2.1 Visual illusions causing SD

Pilots generally use visual cues to help override ambiguous non-visual cues to judge their orientation correctly. But in DVEs, such as a dark night or bad weather, insufficient or misleading visual information can lead to SD (Bos et al., 2003; Sánchez-Tena et al., 2018). *Loss of horizon* – where pilots lose their sense of ‘up’ because of the invisible horizon – is one of the most commonly reported SD experiences due to DVE. This can occur by blowing dust or sand (brown-out), snow (white-out), water (spray-out; due to heavy rain or rotor downwash over water), or atmospheric conditions like when clouds (during high-altitude flights) obscure the horizon. Among RW pilots, restricted visibility due to brown-out, white-out or spray-out is a consistent problem generally during low-altitude flights and landing (Braithwaite et al., 1998a; Gaydos et al., 2012). The lack of stable and reliable visual cues forces pilots to rely on other less reliable visual cues (e.g., clouds) or non-visual cues to judge the gravitational vertical. Pilots can mistake sloping clouds or terrain as the horizon (i.e., *sloping horizon*), misleading their sense of orientation. The conflict between visual (misleading optic flow) and non-visual motion cues (correct vestibular perception of being stationary) can also lead to SD (Pennings et al., 2020).

Distance judgement of objects in the environment is highly dependent on interpretation of various visual depth cues (Brenner and Smeets, 2018; Ooi et al., 2001; Sedgwick, 2021). In aviation, pilots use visual cues such as the trees on the ground or the ground surface texture gradient to judge their altitude (i.e., the aircraft position). Misleading cues (e.g., unexpectedly small trees) or the lack of altitude cues (e.g., featureless ground or smooth calm water) can lead to misjudged altitude, resulting in SD. Such SD is more prominent during a dark night. Dark nights are known to cause the *black hole illusion*, whereby pilots overestimate their altitude, leading them to lower their descent path during the night approach landing (Gibb et al., 2008; Socha et al., 2020). Lack of visual cues due to darkness can also lead to *misjudgment of position in night formation* flying. A common example is a dip illusion where an aircraft trailing behind a lead aircraft unintentionally descends and ‘dips’ below the flight path (Davis et al., 2008; Nakdimon and Gordon, 2025). This can occur while the pilot is trying to keep the lead aircraft at the same spot on the windscreen as the separation between aircraft increases. Nakdimon and Gordon (2025) also reported an opposite case in which an F-15I fighter aircraft approaching an aerial refueling tanker came near-collision because the F-15I pilot did not realize they were climbing while accelerating toward the tanker. Nakdimon and Gordon coined this phenomenon the ‘reverse dip’ illusion.

#### 2.2.2 Body sense (vestibular) illusions causing SD

The ambiguity of vestibular cues (refer to section 1.1) can cause body sense illusions, also referred to as vestibular illusions. **Table 4** lists the names and provides brief descriptions of each vestibular illusion from **Table 3** and their main causes.

**Table 4:** Vestibular illusion descriptions and their main causes.

Name	Brief description	Cause
Undetected drift	Undetected drift motion due to constant velocity or sub-threshold acceleration of the aircraft.	Vestibular equilibrium (otolith organs)
Tumbling sensation (Coriolis illusion)	Tumbling sensation typically felt after a sudden head movement during constant speed turning.	Vestibular equilibrium and Coriolis effect (SCCs)
Graveyard spiral	Sensation of reduced bank angle during prolonged banking, leading to pilot increasing bank angle in an attempt to correct for it.	Sensory adaptation and Aftereffect (otolith organs and/or SCCs)
Graveyard spin	Sensation of rolling in the opposite direction after stopping a prolonged roll manoeuver, leading to pilot start rolling again in an attempt to correct for it.	
Leans	Sensation of tilt after a prolonged banking followed by level flight.	
False sensation of pitching up	Sensation of pitching up due to forward acceleration.	Somatogravic illusion (due to aircraft acceleration; otolith organs)
False sense of pitching down	Sensation of pitching down due to forward deceleration.	
Elevator illusion	Sensation of pitching up due to abrupt vertical acceleration (e.g., updraft).	
False sense of inversion	Sensation of tumbling backward or flying upside down due to abrupt transition from a climb to level flight.	
G-excess	Sensation of tilt due to g-force.	Somatogravic illusion (due to g-force; otolith organs)

During constant velocity or sub-threshold acceleration (i.e., acceleration too small to be detected by the vestibular apparatus; for example, less than 2.5°/second in rotation according to the Mulder’s law from Gradwell and Rainford, 2016), the vestibular system reaches equilibrium (whereby both otolith organs and SCCs are at rest) and does not detect acceleration. Therefore, in the absence of reliable visual cues, pilots may not detect the movement or position of the aircraft relative to the environment resulting in *undetected drift* in RW pilots. An example of this was introduced earlier in the CH147310 Chinook accident (“Flight Safety Investigation Report for CH147310 Chinook accident”, 2024). The report stated that “none of the crew members realized the height and/or rate of descent of the aircraft before impact”.

During the vestibular state of equilibrium (or homeostasis) caused by constant velocity motion, a sudden head movement can cause a cross-coupled stimulation (or *Coriolis* effect) of the pilot’s vestibular system. This effect is primarily sensed by the SCCs (Davis et al., 2008) inducing a tumbling sensation, or a *Coriolis* illusion. This illusion can be induced using the Bárány chair where the trainees are instructed to tilt their heads in various positions (e.g., pitching forward and resting their heads on their hands while seated) during a yaw rotation to stimulate different SCCs. *Coriolis* illusion is one of the leading causes of SD and airsickness for aircrews (Bles, 1998). **Figure 1** illustrates an example of the *Coriolis* effect.

Human sensory organs adapt after prolonged stimulation, leading to reduced sensitivity (i.e., sensory adaptation). For example, during a prolonged bank turn at a constant speed (SCCs at equilibrium), adaptation to the gravitational force cues in the otolith organs results in the aircrews losing the sensation of tilt. In DVEs, where visual cues are unavailable as a reference for aircraft attitude, this adaptation can cause aircrews to think the aircraft is level or not banked enough. An attempt to return to the bank turn by banking the aircraft more, while losing altitude, can lead to the *Graveyard Spiral*.

**Figure 2:**
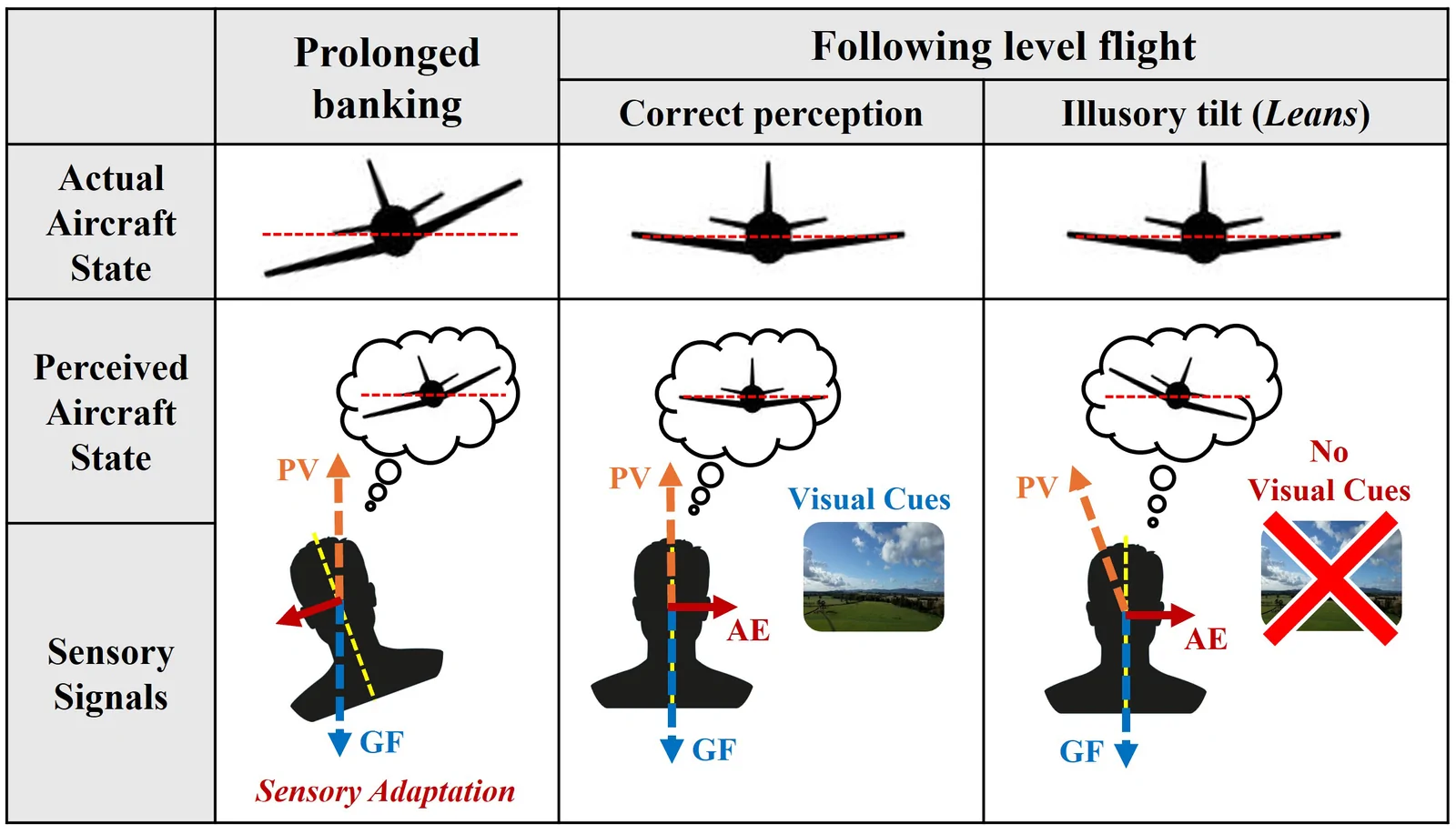
An example of the *leans* illusion. During prolonged banking, a pilot’s otolith organs sensory adapt to the gravitational force (GF). After changing to level flight, the pilot experiences the aftereffect (AE), i.e., the illusion of banking in the opposite direction. The AE alters the pilot’s perceived vertical (PV), no longer aligned with the GF, resulting in the sensation of illusory tilt or *leans*. The red dashed horizontal lines represent the true earth horizontal, and the yellow dashed lines represent the midsagittal plane of the person’s head in the aircraft.

Following vestibular adaptation, the subsequent sensory signals are interpreted as cues in the opposite direction. This is a well-known phenomenon called the aftereffect, shown to impact various human percepts such as visual motion (Addams, 1834; Harris et al., 1981), proprioception (Seizova-Cajic et al., 2007), and vestibular processes (Crane, 2012a, 2012b). Aftereffects in vestibular perception are common causes of vestibular illusions such as a post-roll effect (or a Gillingham illusion), and *leans*. Post-roll effect is a false sensation of rolling back after a roll manoeuver in an aircraft (Crane, 2012b; Ercoline et al., 2000). This illusion can lead to dangerous pilot reactions, like when pilots keep rolling to counteract the false sensation of the aftereffect, known as the *Graveyard Spin*. Another illusion due to aftereffect is *leans* – a false sensation of leaning to the side during level flight – which typically occurs after intentional or unintentional prolonged banking, followed by a quick change to level flight (Holmes et al., 2003). The incorrect expectation of the aircraft attitude due to the *leans* illusion can cause the ‘horizon control reversal’ error where a pilot confuses the aircraft symbol and the horizon symbol on the attitude indicator (Johnson and Roscoe, 1972). This error results in a pilot making an inappropriate rolling manoeuver leading to an SD incident (Landman et al., 2019; van den Hoed et al., 2022). See **Figure 2** for an example of the *leans* illusion after a prolonged banking.

**Figure 3:**
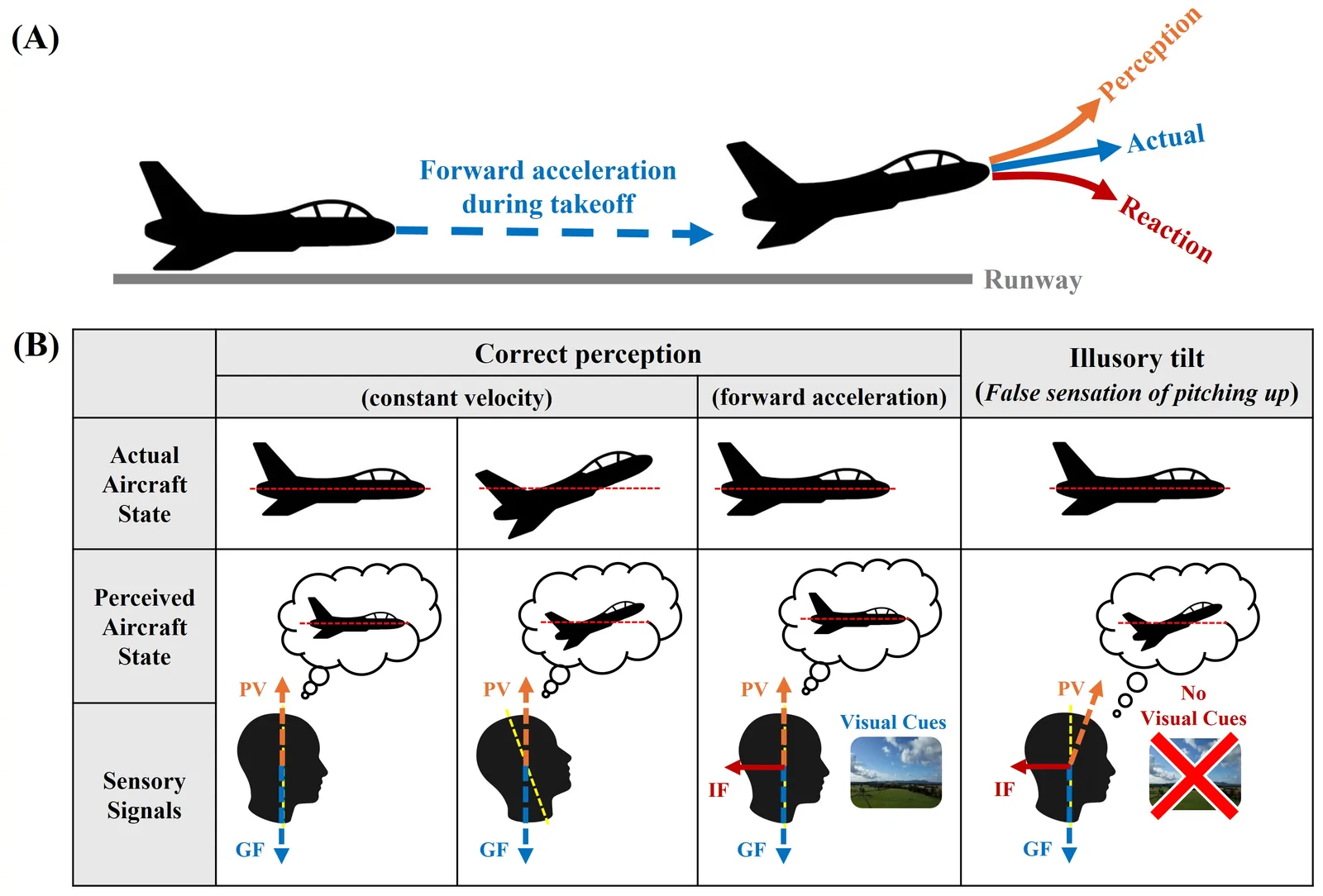
An example of the somatogravic illusion during take-off. **(A)** presents a schematic of an aircraft takeoff. **(B)** illustrates the attitude of the aircraft and the pilot’s head during the takeoff. The forward acceleration induces backward inertial force (IF). When the visual cues (e.g., the horizon) are available, the pilot can correct their perceived vertical (PV) to align with the gravitational force (GF). When the pilot fails to correct their PV, either due to the lack of visual cues or the overwhelmingly extreme sensation of acceleration, IF can alter the pilot’s PV resulting in them experiencing an illusory tilt. In **(B)**, the red dashed horizontal lines represent the true Earth horizontal, and the yellow dashed lines represent the midsagittal plane of the person’s head.

The somatogravic illusion is a false sensation of body tilt due to linear acceleration (Clément et al., 2001; Davis et al., 2008; Groen et al., 2022). Linear acceleration, primarily sensed by the otolith organs, can be interpreted as gravitational force because the vestibular system cannot distinguish the acceleration due to motion from gravitational acceleration (Bos et al., 2003). In aviation, the somatogravic illusion has resulted in pilots confusing forward acceleration with a climb (or the *false sensation of pitching up*) and pitch-over, leading to collisions with ground or water (Gibb et al., 2011). **Figure 3** presents an example of this during take-off. In a more extreme case, when the pilot quickly pitches the nose down during this illusion, the combination of the somatogravic illusion and the change in pitch can make the pilot feel like they are tumbling backward and flying upside down (i.e., the *false sensation of inverse*). Similarly, a quick upward acceleration, typically occurring in an updraft, can also give a sensation of a climb (i.e., *elevator illusion*), leading the pilot to compensate by pitching the aircraft’s nose down and resulting in unintentional descent. During deceleration, pilots have reported the *false sensation of pitching down*.

G*-excess* is another example of a somatogravic illusion involving false, or exaggerated, sensation of tilt due to acceleration during high-g flight (Cheung, 2004; Demir and Aydın, 2021). A pilot can experience an illusory tilt of the aircraft when they move their head, to look at a side panel for example, misaligning their head and the fast-moving aircraft. The high g-force sensed by the otolith organs, interpreted as gravitational force, results in false perception of aircraft tilt. During banking manoeuvers, the false sensation of under-banking or over-banking from *G-excess* can lead the pilot to attempt changing aircraft bank angle.

#### 2.2.3 Other types of SD

In addition to sensory illusions, reliance on flight instruments can sometimes cause SD (see Displays category in **Table 3**). The *roll-reversal error* is an incorrect control input of steering in the opposite direction of the pilot’s intent. This occurs when pilots misinterpret the instruments or when instruments fail, causing the pilot to become disoriented from unexpected aircraft behavior and movement. Head-fixed visual displays such as helmet-mounted displays (HMD) and night vision goggles (NVG) can also limit pilots’ field of view (FOV) and reduce pilot ability to correctly judge aircraft orientation, making pilots more susceptible to SD (Bles, 2008; Braithwaite et al., 1997).

SDs can also occur in the absence of sensory illusions and Display errors (see Other category in **Table 3**). *Poor crew co-ordination* caused by distraction or task saturation can sometimes lead to SD. *Feeling of detachment* is a sense of being physically and psychologically detached from the Earth. Pilots may experience detachment when flying alone at extremely high altitude for extended periods of time. The *Giant hand* illusion is a phenomenon where a pilot feels an invisible force (like a giant hand) pushing the aircraft control. Pilots generally interpret this illusion as a malfunction of aircraft control leading to dangerous overcorrection or ejection from their perceived out-of-control aircraft (Frantis and Petru, 2018; Heinle and Ercoline, 2002; Lyons and Simpson, 1989). These illusions are caused by cognitive factors associated with physical and mental stress. SDs due to these challenges are not well-documented because they are difficult to replicate.

## 3 Approaches to counter SD

Lawson et al. (2017) discussed some traditional SD countermeasures including: clinical selection – pilot selection process to confirm normal functional vestibular responses, human-system interface (HSI) – the hardware and software that enables pilots to interact with the aircraft, and training – pilot education and training for SD familiarization and how to counter it. Clinical selection is no longer used formally because people with healthy functioning vestibular systems are still susceptible to SD (Cheung, 2013; Lawson et al., 2017). In contrast to clinical selection, HSI and training approaches to counter SD are promising but still in development (Lawson et al., 2017). These two approaches to countering SD are discussed in detail below.

### 3.1 Human-system interface approaches to counter SD

Lawson et al. (2017) considered advanced orienting displays (i.e., HSIs that provide aircraft orientation information such as HUD and HMD) as the most promising countermeasures to eliminate SD in human-controlled flight. However, some researchers have argued that new technologies can contribute to SD (Rupert, 2000). While added displays provide more aircraft flight information to the pilot and enhance their situational awareness, the pilot’s visual working memory during flight missions is already at, or near, maximum capacity (Gibb et al., 2016). SD is often unrecognized due to the pilot’s failure to prioritize several competing demands on their attention (Gillingham and Previc, 1993). Crosschecking the information on the displays by verbalizing it to themselves while controlling the aircraft has shown to boost pilot’s visual attention and focus (Kang et al., 2021). This practice is a recommended countermeasure against SD in the Republic of Korea Air Force. But adding more visual information may increase pilot mental workload. For instance, Van Droogenbroeck et al. (2025) found that adding visual pictorial depth cues (e.g., atmospheric haze, shadow and perspective lines) to attitude director indicator increased pilot’s reaction time when rolling back to level from unforeseen bank angles in a motion-base flight simulator. The increased mental workload caused by the additional visual information may lead to task saturation and make pilots more susceptible to SD.

Advancements in aircraft and HSIs allow pilots to operate in more difficult and dangerous environmental conditions. For example, developing and implementing devices like NVG and forward looking infra-red (FLIR) increased the number of operations conducted in nighttime conditions. NVGs also introduce new challenges for pilots’ spatial orientation judgement (Braithwaite, 1997) due to limitations such as decreased FOV, monochromatic image, and reduced visual acuity (Berkley and Martin, 2000; Bles, 2008; Braithwaite et al., 1997; Gwon and Kim, 2006). These limitations, combined with the fatigue from the prolonged exposure to NVG’s degraded visual acuteness and its weight causing neck strain, can lead to higher pilot workload. Cheung (1998) expressed concerns that new challenges from HSIs and their increased use in difficult operations may counteract their intended improvement to reduce SD incidence. According to a USAF report by Sundstorm (2004), more than half of reported SD mishaps during night flights were caused by NVG use. Braithwaite et al. (1998b) also reported that NVGs were involved in 43% of all SD-related accidents in helicopter flight records from 1987-1995, but only in 13% of non-SD accidents. This demonstrates a potential association between NVG use and SD. These findings validate Cheung’s concerns and emphasize the importance of proper SD training with new HSIs such as NVGs for aircrews.

Using non-visual cues such as auditory and tactile alerts in addition to visual displays have shown some potential as effective SD countermeasures and may be suitable to support pilots in recovery from SD (Daiker et al., 2020; Paillard et al., 2014; Rochlis and Newman, 2000; Rupert, 2000; van Erp, 2007). Auditory warnings (e.g., aural cues providing alerts for deviations from the recommended flight path) can reduce pilot workload and reaction time during a flight when properly designed (Paillard et al., 2014). However, when applied poorly, auditory warnings provide no benefit (McAtee et al., 2017) or may even distract pilots and degrade flight performance (Russell et al., 2016).

*Three-dimensional* (3D) audio providing directional cues (e.g., the direction of Earth’s gravity) are also actively being investigated to counter SD. But studies with 3D audio have shown inconsistent results in people’s ability to accurately localize the auditory cue’s sources (Brill et al., 2016) and requires further research. Brill et al. (2016) instead recommended combining 3D audio with vibrotactile cueing to improve its spatial accuracy. Combining aural cueing with a Tactile Situation Awareness System (TSAS) – providing speed, drift, and altitude control cues – has shown synergetic benefits in some RW manoeuvers (Russell et al., 2016).

Wearable suits with TSAS have also been in development for decades (Gibb et al., 2011; Rupert, 2000; Rupert et al., 2023, 2016; Schultz et al., 2009). Rupert (2000) reported that with training, RW and fixed-wing (FW) aircraft pilots could rely on haptic cues from TSAS on their torso for attitude information on aircraft pitch and roll angles. TSAS improved performance in RW hover and landing manoeuvers during DVE (Kelley et al., 2013; Schultz et al., 2009) even in sleep-deprived pilots (Curry et al., 2008). Rochlis and Newman (2000) developed a tactor locator system (TLS) for astronauts, using tactors on their neck and torso, which provided position and velocity information of a target in a computer simulation of the International Space Station (ISS). They found that the TLS decreased reaction and movement time of the astronaut in target pursuit tasks. Vibrotactile feedback about body orientation has also shown to improve performance in a balancing task, simulating dynamic orientations in spaceflight, with sufficient experience and training (Vimal et al., 2025, 2023). However, any development of new technology-based SD countermeasures is subject to rigorous Technology Readiness Level assessments (Cheung, 2017). SD countermeasures utilizing these non-visual cues, to our knowledge, are not yet commonly deployed for use by pilots (Lawson et al., 2016).

Kim et al. (2023) tested galvanic vestibular stimulation (GVS) – a technique of applying small electrical currents on the mastoid processes behind each ear to stimulate vestibular responses – in a motion-based simulator exposing pilots to SD-inducing flight manoeuvers. They found that applying a low-current GVS (± 2 mA) can possibly counteract the *leans* illusion in pilots during the manoeuvers. But GVS can translate into motion cues indicating to the user that they are tilting left or right and, therefore, using it in a real flight is still risky. Moreover, in another study using GVS on non-pilots by Pu et al. (2012), vestibular signals from GVS resulted in reduced ability to maintain arm and hand position which may have implications for pilots maintaining hand position on aircraft controls. Dilda et al. (2012) also found that GVS negatively influenced some cognitive tasks. These negative effects may lead to impaired aircraft operations and must be carefully evaluated before considering GVS as a suitable in-flight SD countermeasure.

### 3.2 Training approaches to counter SD

SD incidents have been traditionally reported as Type I, Type II and Type III SD. Type I SD is when the pilot is unaware of the SD. Type II SD is when the pilot is aware of the SD. Type III SD is an incapacitating SD where the pilot is unable to reorient themselves physically, psychologically or both, often associated with high anxiety and fear (Bles, 2008; Heinle and Ercoline, 2002). A typical SD incident occurs in stages, starting as Type I, then progressing to Type II, and in rare cases progressing to Type III (Dixon et al., 2026). Type III SD is difficult to predict as it is not well-understood (Heinle and Ercoline, 2002). This is because any Type I or II SD can develop into Type III SD (Bles, 2008). Training to overcome or manage SD is only useful in the case of Type II SD. Hence, most SD accidents occur from pilot reaction to Type I SD when they are unaware that they are disoriented. Pilots are trained to rely on instruments instead of their ‘gut’ for this reason. Dixon et al. (2026) suggested that the pilot’s anticipation of SD stimuli during a flight, learnt from training and education, can eliminate the Type I stage (refer to Figure 3 in Dixon et al., 2026). Therefore, the goal of SD training programs has been to convert Type I SD incidents to Type II SD incidents through education and SD demonstrations (Heinle and Ercoline, 2002).

Over the decades, experts have made various recommendations to improve SD training procedures (e.g., Cheung, 1998; Estrada et al., 2003; Gibb et al., 2011; Lawson et al., 2017; Walker et al., 2009). For example, Walker et al. (2009) surveyed 21 subject matter experts (SMEs) in SD (e.g., experienced aviators and scientists studying SD) and many of the respondents believed physical motion was necessary for SD training. Cheung (2013) argued that SD training should be implemented in student flight training curricula. A list summarizing some SD training improvement recommendations from the past literature is presented below:

1. Recognize the limitations of technologies such as NVG that may increase pilots’ susceptibility to SD (Cheung, 2013). Educate and train the pilots with these limitations in mind.
2. Combine ground-based training, like classroom lectures, with demonstrations and exposure to SD using advanced simulation devices with motion cues (Boril et al., 2018; Cheung, 1998; Gibb et al., 2011; Lawson et al., 2017; Walker et al., 2009).
  • SD training programs should include in-flight SD exposure by a dedicated sortie operated by Instructor Pilots (IPs; Cheung, 1998; Gibb et al., 2011) while students are flying. The goal is to experience SD, recognize, and recover from it (Lawson et al., 2017) under IP supervision. For example, in a real sortie with a trainee and an IP, the *leans* illusion can be reproduced by doing a prolonged bank turn followed by level flight.
3. Provide regular (e.g., annual) SD refresher training for the pilots (Boril et al., 2018; Estrada et al., 2003; Lawson et al., 2017).
4. Extend SD training and demonstrations beyond pilots to also include aircrew (Lawson et al., 2017). Crew coordination is an important aspect that can induce or prevent SD. Aircrews familiar with SD may recognize it before the pilot and can alert them.
5. Develop new SD training effectiveness evaluation methods to assess current and future SD training (Cheung, 2013). For example, tracking SD mishaps annually to evaluate the effectiveness of the SD training conducted each year.

Although not related to SD training, Cheung (1998) also urged practitioners to consider the risks of SD specific for each mission during the mission risk analysis. Such practice can reduce the risk of SD due to poor crew coordination during the mission.

#### 3.2.1 Examples of SD training methods used across militaries

Despite recommendations to improve SD training, only a few have been implemented into official SD training programs. Lawson et al. (2017) listed the state of SD training in various countries, inferred from a 2016 Air and Space Interoperability Council Report, and compared them to past recommendations. **Table 5** shows the SD countermeasure training in some countries, based on the report as of 2006 (Bles, 2008) with some updates from 2016 (Lawson et al., 2017).

**Table 5:** SD training in various militaries (Bles, 2008; Lawson et al., 2017).

Type of SD training	Canada RCAF	US Air Force	US Navy	US Army	UK RAF	New Zealand RNZAF	Australia RAAF
Classroom	LS	LS, BC	LS	LS	LS	LS	LS, NVG
Ground Based	BC, GI	BC	BC*, MSDD	BC	DISO	BC	BC, GI
Flight Sim Based		NVG	No	No	Various		No
In-Flight SD Demo	No*	Yes	No	Yes	Yes*	Yes*	Yes*
In-Flight to Overcome or Manage SD	UAT	INR	UAT	UAT	UAT	UAT	UAT*
Refresher Training Frequency	5 years	5 years	4 years	1 year	5 years	5 years	3 years
Measurement of Training Effectiveness	Written exam	Class A mishap tracking	Written exam	Written exam	Surveys and mishap tracking	Written exam	Written exam

LS, Lecture series; BC, Bárány Chair; NVG, Night Vision Goggle; GI, Gyro IPT; MSDD, Multi Station Disorientation Demonstrator; DISO, AIRFOX DISO Spatial Disorientation Trainer; UAT, Unusual Attitude Recovery Training; INR, In-Flight Recovery.

The empty cells mean there was no information available in the reports.

*Updates from Lawson et al., 2017.

As shown in **Table 5**, there was virtually no change in SD training programs in the 10 years between the reports from Bles (2008) and Lawson et al. (2017). This is discouraging considering that numerous past studies confirm training is an effective SD countermeasure. The Bárány chair is the most commonly used apparatus for the ground-based SD demonstrations by militaries (refer to **Table 5**). But it can only stimulate the SCCs in a single axis at a time which is not representative of real flight. To faithfully reproduce in-flight vestibular percepts in ground-based simulation, more sophisticated SD simulation devices are needed. To address this, motion-based SD simulators have been developed and implemented into SD training programs over the years. Section 3.2.2 describes the outcomes of these SD simulator training and in-flight SD demonstrations.

#### 3.2.2 Ground-based simulator and in-flight SD demonstrations

In exposure training approaches to countering SD, Previc et al. (2007) showed that older pilots (i.e., with more flight hours) are better at detecting SD events in a simulator than younger pilots (i.e., with less flight hours). But other research does not indicate a clear relationship between flight hours and SD susceptibility (e.g., Lewkowicz et al., 2020; Socha et al., 2026; van den Hoed et al., 2022). This may be because pilots all have unique flight experiences and so more flight hours do not necessarily predict more *SD experience.* In addition, dedicated SD exposure training has shown to possibly mitigate cognitive impairment during SD (e.g., Gresty and Golding, 2009; Gresty et al., 2008), possibly demonstrating the importance of ground-based SD exposure training.

The efficacy of simulation-based SD demonstrations in SD training has been perceived favorably according to pilot surveys from the RAF (Bushby and Gaydos, 2023; Powell-Dunford et al., 2016), U.S. Army (Johnson et al., 1999), Indian Air Force (Baijal et al., 2006), RNLAF (Pennings et al., 2020) and among civilian flight instructors (Karapetjan et al., 2022). However, these subjective findings require validation from empirical research studies. Among the papers reviewed here, only the Polish Air Force Institute of Aviation Medicine (PAFIAM) reported research-based evaluation of their SD counter measures program (Kowalczuk et al., 2002). In this program, pilots first underwent a series of lectures on SD. Then they were trained in a flight simulator capable of generating visual illusions leading to SD. Kowalczuk et al. tracked pilots’ reaction and eye movements to detect SD incidents during flight simulator-based training. SD incidents were detected by noting an increase in intensity of saccadic eye movements. Type I SD was identified by no aircraft control counteraction from the pilot during those SD incidents, consistent with the pilot being unaware of SD. Type II SD was identified by pilot’s counteraction to SD. SD symptoms were detected in 60% of all flights: 20% of SD incidents were Type I and 40% were Type II. While these results were not compared to other methods to evaluate the new course’s effectiveness, monitoring and comparing Type I and II SDs and associated eye movement data in flight manoeuvers was novel and can potentially be used as a method to evaluate SD training effectiveness. Later, PAFIAM used a simulator that can produce angular accelerations in three axes simultaneously, called the Gyro Integrated Physiological Trainer (IPT), adding the capacity to create vestibular SD illusions in addition to visual SD illusions to their SD training program. The IPT can also expose pilots to SD while they try to maintain full control of the aircraft before, during, and after SD (Bogusz, 2025). Kallus et al. (2011) showed that pilots trained on a motion-base simulator similar to the IPT performed better in SD recovery tests than pilots trained without motion. Kallus et al. also found evidence of possible negative transfer of training with no-motion training, highlighting the importance of motion cues in SD training. A human-rated centrifuge – a rotating device used to simulate high-g forces on the human body – is also capable of inducing SD and was actively considered for SD demonstrations and training by the PAFIAM, according to Kowalczuk et al. (2002). But we found no report of a centrifuge being implemented into any SD training programs.

The British Army Air Corps organized an SD sortie program for RW flight training in 1982 with refresher sorties flown every 4 years thereafter (Braithwaite, 1997; Hiatt and Braithwaite, 2003). The SD accidents compared between the period before (1971-1982) and after (1983-1993) the introduction of dedicated SD sortie showed significant SD accident rate reduction. The rate decreased from 2.04 (pre-1983) to 0.57 (post-1983) per 100,000 flying hours. While there are confounding factors that contributed to this analysis (e.g., installation of additional flight instruments and increase in the use of NVG), the reduced number of SD accidents is encouraging.

## 4 Discussion and recommendations for future research

Based on the research presented thus far in this review, we note two major gaps in the SD literature and its countermeasures. First, the existing SD and training literature are limited. Despite expert recommendations of increasing research in SD (e.g., Cheung, 2013; Lawson et al., 2017), most of the literature found was on subjective data (e.g., surveys) rather than objective experimental results. Second, modern technologies such as virtual reality (VR) head-mounted displays and artificial methods to generate self-motion/disorientation in the vestibular system have not been explored as potential SD mitigation tools in the literature. In-flight training is the most effective method to demonstrate SD for aircrews, as it provides trainees with the SD experience in aviation most faithfully and is highly recommended by numerous experts (for example, Cheung, 1998; Gibb et al., 2011; Lawson et al., 2017). But running a dedicated SD demonstration sortie is costly and poses a risk of accidents. Exploring new technologies for developing safe and efficient ground-based SD demonstration techniques will be beneficial to the aviation community.

### 4.1 Vestibular stimulation methods

Artificial vestibular stimulation methods have been shown to induce illusory self-motion in the vestibular system without the need to physically move a person (Kirollos and Herdman, 2023a; Lim and Kim, 2025; Pradhan et al., 2022b; Wagner et al., 2025). Specifically, caloric vestibular stimulation (CVS) – a method using warm or cold water/air in the ear canal to stimulate the vestibular system – has been shown to produce yaw-axis illusory self-rotation. Kirollos and Herdman (2023a) demonstrated that cold air CVS cooling the endolymph fluid of the horizontal SCC, can induce vestibular circular vection for periods greater than 45 seconds. GVS can generate a tilting percept in all three axes (pitch, yaw, and roll). Synchronizing GVS with visual stimuli in VR has been shown to enhance perception of self-motion (Wagner et al., 2025) and mitigate visual-vestibular conflict (Pradhan et al., 2022b). Lim and Kim (2025) suggested that bone conduction (BC) vestibular stimulation – a method of vibrating the skull at frequencies that activate the inner ear’s vestibular organs – may be able to generate directionally accurate perception of linear self-motion (< 6° deviation) and yaw axis self-rotation in a computational model. Some past studies suggested that BC vibration can stimulate the otolith organs in guineapigs (Curthoys et al., 2006) and in humans (Weech and Troje, 2017). But whether it can induce self-motion perception must be verified.

Although there is a lack of studies investigating BC and CVS as potential solutions for SD demonstrations, GVS has been suggested to disorient pilots in a safe environment to familiarize them to SD (Allred et al., 2024; Pu et al., 2012). Past studies using GVS to induce SD illusion, however, showed mixed results. For instance, Pradhan et al. (2022a) found that applying mismatched GVS during VR-based flight simulation enhanced SD illusion during simulated take-off (somatogravic illusion) and sustained turn (*Coriolis* illusion). But Houben et al. (2024) found that less than one third of participants (total N = 14) they exposed to GVS-induced roll, in a fixed-base flight simulator, experienced post-roll illusion afterwards. These findings demonstrate the challenges in using GVS to induce specific SD illusions and highlight the need for more research on this topic. Combining GVS with other vestibular stimulation (e.g., the CVS) could widen the range and the variety of artificial vestibular stimulation profiles. In a review article, Allred et al. (2024) suggested combining GVS with linear acceleration (e.g., gravity or real motion) to accommodate its limitations, but these combinations have not been previously tested.

A tangible research approach to implement vestibular stimulation into SD training would be to focus on developing techniques to demonstrate common illusions such as *leans* and *Coriolis*. These illusions are generally demonstrated using the Bárány chair. But this spinning chair is limited to producing yaw rotation along the vertical axis only, stimulating the canals in a single axis at a time. Combining artificial vestibular stimulation methods with motion chairs, such as the Bárány chair, adds realistic range in possible head motion sensations and resulting illusions. This would allow SD demonstration without risk and cost associated with using an aircraft. Researchers, in consultation with SMEs, must identify the real flight scenarios that can lead to each type of SD for the aircrews in both FW and RW aircraft. These situations should be simulated faithfully in demonstrations utilizing the vestibular stimulation methods described here.

Researchers must also consider the possible long-term effect of these vestibular stimulations on the trainees. Cheung (1998) warned that training may cause unintentional negative transfer of training. This can result from undesirable adaptation of learned habits during training, but it can also occur from the long-term exposure to a sensory stimulation. For example, a long-term adaptation to visual (from optic flow) or vestibular (from GVS) stimulations enhancing or inhibiting sensation of body sway motion has been shown to temporarily alter the weights of visual and vestibular senses in people’s postural control (Kitazaki and Kimura, 2010). The possible influence of such negative transfer effects and their outcomes on aircrews requires rigorous validation.

### 4.2 SD demonstration in centrifuge and motion platforms

The high acceleration involved with current generation aircraft (up to 9 G) cannot be accurately reproduced in a typical flight simulator or part-task trainer. Such high-g acceleration needs to be conducted in-flight or using a centrifuge. In the past, SD illusion demonstrations related to high-g (i.e., somatogravic and *G-excess* illusions) was not considered an essential part of training for either FW or RW aircraft (Bles, 2008). But Lawson et al. (2017) pointed out that the symptoms typically associated with *Coriolis* effect can also occur due to the *G-excess* effect, mostly in FW aircraft. Given that *G-excess* has been detected at forces as low as 1.3 G during RW manoeuvres (Baylor et al., 1992), Lawson et al. (2017) argued that *G-excess* should be demonstrated for both FW and RW pilots in a human centrifuge. A human-rated centrifuge is suitable to expose aircrews to high-g (Kim et al., 2025). Future research should investigate whether centrifuge training can help counter *G-excess* illusions.

Human centrifuge may be used for aircrew training to provide SD familiarization. Centrifuge-based spatial orientation training with sensory feedback (e.g., visual, auditory, and haptic cues providing orientation information) has shown to improve people’s perception of g-load in pilots (Brink et al., 2024) and non-pilots (Keramidas et al., 2024). Instructing pilots to attend to the SCCs inputs also improved their perception of bank angle at 60°. VR displays paired with centrifuge or other vestibular stimulation methods (e.g., motion chair) may also be a potential solution to demonstrate g-related SD illusions effectively. For example, VR head-mounted displays combined with a motion chair platform has shown to effectively induce some SD illusions such as *Coriolis*, *leans*, *graveyard spiral*, *false horizon*, *black hole* and somatogravic illusions (Kim et al., 2025; Thomas et al., 2023). But combining VR with a human centrifuge has not been attempted to our knowledge. In addition to SD demonstrations, the researchers can also investigate the onset time, duration, and the threshold or range of g-force where the linear acceleration causes SD. The findings from this proposed research may help in developing more fine-tuned SD scenario simulations and new training methods for the aviation community and air forces. A typical human centrifuge used for pilot training consists of a gondola – a capsule at the end of the rotating arm where a person sits inside. As the gondola accelerates, it can become parallel with the Earth’s horizontal axis with increasing speed. To faithfully reproduce vestibular signals associated with SD such as *G-excess* or somatogravic illusions, the angle and the intensity of the g-load experienced by the person sitting in the centrifuge must be controlled. Adding the capability to adjust the person’s sitting orientation inside the gondola or fixing the gondola tilt angle would open a large variety of options for SD research. Similarly, hybrid centrifuge-based SD training simulators with a cabin mounted on a gimbal suspension (allowing for additional rotational degrees-of-freedom; roll, pitch and yaw) have also been suggested as SD demonstration tools (Alexandrov et al., 2025; Kvrgic et al., 2015; Winter et al., 2023).

### 4.3 SD training effectiveness evaluation

To evaluate the effectiveness of SD training methods recommended so far, a method to reliably and objectively evaluate the trainees’ understanding and ability to recognize SD must be established. Exposing pilots to various motion-based SD events in simulations, such as in Boril et al. (2020); Kowalczuk et al. (2002) and Kim et al. (2025), may be a safe and cost-effective option to test whether they can recognize and counter SD after their training. In the simulations, pilots can perform a flying task during or following a flight profile inducing SD. In addition to subjective self-reports, objective measures should be used to index and validate SD experience during the simulations. Possible measures to objectively index SD from the literature include: postural sway (Hao et al., 2022), electrodermal activity (Tamura et al., 2018), electroencephalography (Feltman et al., 2026; Geva et al., 2025; Hao et al., 2020; Rouser et al., 2026), eye-tracking and cardiovascular responses (Cheung et al., 2004; Ledegang and Groen, 2018). The results from these objective measures can be cross-validated with the subjective reports from the trainees. Identifying which SDs are unrecognized by pilots would reveal weaknesses in the current SD training and provide insights to improving training programs in the future. However, these potential objective SD measures require further research and validation.

## 5 Summary of recommendations

In summary, researchers should focus on developing techniques to generate SD inducing illusions effectively and efficiently to expose aircrews to them in training. An objective method to evaluate SD training effectiveness also needs to be developed. We recommend the following for future research on SD countermeasures.

- Identify airborne manoeuvers or missions that commonly induce SD illusions in FW and RW pilots.
  i. Aviation SMEs should be consulted in identifying these potential SD-inducing scenarios and their characteristics (i.e., parameters).
  ii. The important parameters for these SD scenarios should include: magnitude and direction of acceleration, aircraft bank angles, head motions, and environmental visual conditions.
- Develop techniques to demonstrate various SD-inducing illusions faithfully.
   i. Using the parameters identified in (a), researchers should develop techniques to simulate SD illusions utilizing tools such as: SD simulator, motion chair, VR displays, human centrifuge and artificial vestibular stimulation.
   ii. Combining more than one tool (e.g., artificial vestibular stimulation with motion chairs or VR displays with human centrifuge) can increase the variety of SD illusions that can be demonstrated.
   iii. Human centrifuges may also be used to demonstrate SD illusions due to high accelerations and g-forces they can attain (i.e., G-excess and somatogravic illusions).
- SD training effectiveness evaluation methods with objective measures must be established.
   i. The frequency count of Type I compared with Type II SDs in SD exposures may be an objective measure for evaluating training effectiveness.
   ii. After training, the trainees can be exposed to SD scenarios, using the techniques developed in (b), followed by a post-scenario survey on SD detection.
   iii. During the exposure, the trainees’ eye movements, physiological responses, and behaviors should be monitored and recorded.
   iv. The trainee reactions during the SD scenario, both physiological and behavioral, can be used to evaluate the trainee’s ability to recognize SD. The evaluation from the trainee reactions can be cross-validated with the post-scenario survey answers.

## 6 Limitations

The major limitation of this narrative review was that the literature search did not follow a strict, reproducible protocol. This limitation may have restricted the size of the literature corpus. The predominantly western military context of the literature available and the focus on the military aviation (i.e., limited representation from civilian aviation) may have also introduced bias in the sources selected.
